# The *Trypanosoma cruzi* Protease Cruzain Mediates Immune Evasion

**DOI:** 10.1371/journal.ppat.1002139

**Published:** 2011-09-01

**Authors:** Patricia S. Doyle, Yuan M. Zhou, Ivy Hsieh, Doron C. Greenbaum, James H. McKerrow, Juan C. Engel

**Affiliations:** Tropical Disease Research Unit and Sandler Center for Drug Discovery, Department of Pathology, University of California, San Francisco, California, United States of America; Imperial College London, United Kingdom

## Abstract

*Trypanosoma cruzi* is the causative agent of Chagas' disease. Novel chemotherapy with the drug K11777 targets the major cysteine protease cruzain and disrupts amastigote intracellular development. Nevertheless, the biological role of the protease in infection and pathogenesis remains unclear as cruzain gene knockout failed due to genetic redundancy. A role for the *T. cruzi* cysteine protease cruzain in immune evasion was elucidated in a comparative study of parental wild type- and cruzain-deficient parasites. Wild type *T. cruzi* did not activate host macrophages during early infection (<60 min) and no increase in ∼P iκB was detected. The signaling factor NF-κB P65 colocalized with cruzain on the cell surface of intracellular wild type parasites, and was proteolytically cleaved. No significant IL-12 expression occurred in macrophages infected with wild type *T. cruzi* and treated with LPS and BFA, confirming impairment of macrophage activation pathways. In contrast, cruzain-deficient parasites induced macrophage activation, detectable iκB phosphorylation, and nuclear NF-κB P65 localization. These parasites were unable to develop intracellularly and survive within macrophages. IL 12 expression levels in macrophages infected with cruzain-deficient *T. cruzi* were comparable to LPS activated controls. Thus cruzain hinders macrophage activation during the early (<60 min) stages of infection, by interruption of the NF-κB P65 mediated signaling pathway. These early events allow *T. cruzi* survival and replication, and may lead to the spread of infection in acute Chagas' disease.

## Author Summary

*Trypanosoma cruzi* (*T. cruzi*) is the unicellular parasite that causes Chagas' disease, a devastating health burden throughout Latin America now also affecting developed countries. Macrophages are the first cells that become infected by *T. cruzi* and disseminate the infection to other tissues. The parasite then preferentially infects and multiplies within heart muscle cells causing severe heart disease and often death. The new drug K11777 targets a vital parasite enzyme, the protease cruzain. Consequently, it is important to understand what the enzyme is doing during infection. To elucidate the role of the protease, we compared infection of macrophages with parental wild type parasites and with protease deficient *T. cruzi*. We now report a role for the parasitic protease in immune evasion. The protease prevents macrophage activation thus allowing *T. cruzi* survival and replication, and favoring the spread of infection.

## Introduction

*Trypanosoma cruzi* is the parasitic agent of Chagas' disease that affects approximately 12 million people throughout Latin America (WHO). Current chemotherapy with nifurtimox and benznidazole is unsatisfactory due to severe side effects that require medical supervision [1]–[3]. *T. cruzi* infection is classically transmitted by an insect vector, the reduviid bug [4]. Parasites disseminate from the insect bite site and, in the most common clinical course of Chagas' disease, infect cardiac myocytes leading to acute myocarditis or chronic infection with relentless cardiac failure. Fulminant disease is commonly seen with HIV infection or immunosuppression. In patients with immunodeficieny, parasites may be found in many organs, and a highly fatal meningoencephalitis often ensues. These observations led us to hypothesize that *T. cruzi* successfully evades the host immune response, and may in fact utilize unresponsive macrophages as a means of egress from the insect bite site prior to dissemination to other cell types [5]. While the specific mechanisms of immune evasion by *T. cruzi* remain largely unknown, several reports have suggested that the major protease of *T. cruzi*, cruzain, (*a.k.a.* cruzipain, GP57/51) is a key factor [6], [7].

Cruzain plays a biological role in cell remodeling during transformation of the insect epimastigote stage of *T. cruzi* to infectious metacyclic [8]–[11]. Scharfstein and collaborators found cruzain involvement during trypomastigote infection by the proteolytic release of kinin from host cells surfaces and activation of bradykinin receptors [12]–[14]. More recent reports suggested that cruzain mediates anti-apoptotic mechanisms in *T. cruzi*-infected myocardiocytes *in vitro* [15]. The biological role of cruzain in the intracellular amastigote stage of *T. cruzi* and in Chagas' disease pathogenesis remains nevertheless largely unknown. Assessments of the role of cruzain in *T. cruzi* pathogenicity have been hampered by genetic redundancy and the failure of gene deletion attempts [9], [12], [16], [17]. Successful knockout of the cruzain gene cluster has not been achieved to date leading to speculation that deletion of the cruzain coding genes might be lethal. Chemical knockout with cysteine protease inhibitors was also lethal for *T. cruzi* [18]. Although the role of auto-proteolysis in cruzain activation was confirmed, these studies did not allow elucidation of the biological role of this protease in human disease. Duschak *et al.* [19], [20] observed lower cruzain activity and protease sequence alterations in attenuated strains as compared to their virulent parental *T. cruzi,* while other authors found infectivity is not dependent on cruzain expression or activity [21]. An alternative approach to study the biological role of this vital gene product is to examine the phenotype of protease-deficient organisms. We generated a protease deficient *T. cruzi* that retained less than 1% of cruzain activity of the wild type parental clone [22]. We now report that cruzain deficient *T. cruzi* rapidly activate host macrophages via NF-κB P65 and are unable to survive intracellularly within macrophages. In contrast, infection with wild type parasites appears to induce cruzain-mediated proteolysis of NF-κB P65 leading to unresponsiveness of the host macrophage during early (<60 minutes) infection. This immune evasion mechanism may be critical for *T. cruzi* survival during early natural infection with a low number of trypmastigotes.

## Materials and Methods

### Parasites and cells

Wild type CA-I/72 *T. cruzi* was isolated from an Argentinean chronic chagasic patient and cloned [23]. Cruzain-deficient, and cysteine protease inhibitor (K11777)-resistant (CA-I/KR; KR) *T. cruzi* were derived from parental CA-I/72 parasites [22]. The cysteine protease inhibitor K11777 (K777, N-Methyl-Pip-F-hF-VSΦ) was kindly provided by J. Palmer, Celera, CA [18], [24]. Epimastigotes were maintained at 26°C in Brain-heart tryptose (BHT) medium [25] with 10% heat inactivated fetal calf serum (FCS), and with the addition of 20-fold the lethal dose (200 µM) of K11777 for phenotypic cruzain-deficient CA-I/KR.

Bovine embryo skeletal muscle cells (BESM) cells were a kind gift of J. A. Dvorak, NIH. Mammalian stages of wild type (WT) CA-I/72 were maintained in BESM cells with RPMI-1640 medium and 5% heat inactivated horse serum (HS) at 37°C [26]. Intracellular cruzain-deficient CA-I/KR *T. cruzi* were maintained serially in BESM as above but with the addition of 5% heat inactivated FCS, 5% heat inactivated horse serum, and 10 µM K11777. Long-term culture at higher concentrations of the inhibitor was toxic for BESM cells. For some experiments, the mammalian stages of cruzain-deficient CAI/KR were also cultured for 4 passages (2 months) in the absence of inhibitor. The approximate duration of the intracellular cycle of wild type CA-I/72 (4.5 days) [23] and CA-I/KR (18 days) was estimated in infected BESM cells by contrast phase microscopy and by fluorescence microscopy of propidium iodine stained slides. Cruzain-deficient parasites were unable to survive and develop within macrophages.

J774 mouse macrophages were from the UCSF cell culture facility. J774 cells were cultured as above and in some experiments irradiated (1000 Rad) 24 h prior to use to arrest cell division. Normal peritoneal macrophages were collected from C3H mice (The Jackson Lab).

### Ethics statement

This study was carried out in strict accordance with the Guide for the Care and Use of Laboratory Animals of the National Institutes of Health. The protocol was approved by the Institutional Animal Care and Use Committee of the University of California, San Francisco (AN079928-02A).

### Protease labeling

WT and cruzain-deficient epimastigotes were washed three times in PBS, counted in a Coulter Counter Multisizer 3 (Beckman) and taken to equal numbers of parasites/ml, lysed by 5 cycles of freeze-thawing, centrifuged at 14,000 *xg* for 1 h at 4°C [22], and frozen at −70°C until used. Fractions (10^5^ epimastigotes/lane) were reacted with the iodinated inhibitor DCG04 [27], [28] and electrophoresed. Resolved molecular species were then isolated and analyzed by nano LC/MS, CID spectra, and/or fractionation by MALDI-TOF at the Protein Facility, UCSF. Protein fragments (9-17 aa long) were analyzed by NCBI blast.

### Immunoelectron microscopy (IEM)

BESM cells infected with intracellular amastigotes of WT or cruzain-deficient *T. cruzi* were processed for IEM with a specific anti-cruzain polyclonal antibody as previously described [24]. Briefly, *T. cruzi*-infected BESM cells were collected by centrifugation, washed twice with PBS and fixed for 2 hours at 4°C with 2% paraformaldehyde-0.05% glutaraldehyde in 0.1 M phosphate buffer, pH 7.4. Cells were then cryoprotected, frozen, sectioned, and immunolabeled sequentially with rabbit polyclonal anti-cruzain Ab and goat anti-rabbit IgG-10 nm gold-labeled Ab (dilutions 1∶250 and 1∶500, respectively). Thin sections were observed in a Tecnai 10 (FEI Co.) electron microscope. Gold-labeled cruzain localizing to the amastigote cell surface was quantified in micrographs of intracellular parasites in three independent experiments (n = 55 wild type amastigotes; n = 55 cruzain-deficient amastigotes). The cell perimeter of sectioned intracellular amastigotes was measured with Openlab software (Improvision), and cruzain density was expressed as the number of gold labeled cruzain particles/µm of parasitic cell membrane. Results were statistically analyzed (t test).

### Western blots (WB)

J774 macrophages were seeded onto 12 well tissue culture plates for 24 h prior to infection with WT or cruzain-deficient *T. cruzi* as appropriate. Infection was performed at a low ratio of 0.5 parasites/macrophage. Some infected cultures were treated with cysteine protease inhibitor. Controls consisted of J774 macrophage cultures that were uninfected, treated with purified LPS (150 ng/ml LPS, Sigma) [29], or treated with K11777. Samples were collected at 1 min, 30 min, 60 min, and 150 min, and at 48 h post-infection. Monolayers were washed twice with cold PBS, solubilized in 1 ml cold PBS with1% Triton X-100, scraped, aliquoted, immediately snap-frozen, and stored at −70°C until used. Samples were centrifuged at 14,000 xg for 30 min at 4°C and supernatants taken to identical protein concentration. Samples (1 mg protein/lane) were heated at 70°C for 5 min and resolved by Nupage electrophoresis (10% BisTris gels at 200 V, Mops Buffer) (Novex, Invitrogen) prior to transferring to PVDF membranes (35 V for 2 h). Blots were blocked overnight with 1% BSA (Sigma). Samples were blotted with rabbit anti–NF-κB P65 Ab, rabbit anti- iκB Ab, mouse anti-phosphorylated (∼P) iκB Ab (Santa Cruz Biotech, CA) and anti actin Ab (Cal Biochem). All methods were according to manufacturer's instructions (Santa Cruz Biotech, CA). Additional controls were similarly infected with *Leishmania mexicana*, a kind gift of J. Mottram (University of Glasgow, UK), for 48 hours. Results were confirmed in duplicate experiments according to methods described by Ma and colleagues [30]–[32]. Briefly, 5×10^6^ cells per point (1, 10, 30, and 60 min) were infected with *T. cruzi* trypomastigotes as above (ratio 0.5 parasites/cell). Non-infected macrophages either untreated (MΦ) or treated with LPS, and/or K11777 as indicated (10, 20, 40 and 60 min) were also used as controls. Macrophages were washed twice with PBS, scraped, transferred to eppendorf tubes, and centrifuged prior to lysis with NP40 lysis buffer (0.1% NP40, 1 mM Na vanadate and 5 mM Na fluoride−50 mM HEPES). Nuclei were then pelleted by centrifugation (20 min at 14,000 *xg*, 4°C) and discarded, and the supernatants were boiled in Laemli buffer for 10 min immediately prior to SDS-PAGE/WB (Cell Signalling Technology) [30], [31]. Membranes were developed sequentially with anti ∼PiκB Ab (Cell Signalling), anti-iκB Ab (Cell Signalling), and anti-actin Ab (Southern Biotech) [30].

### Confocal microscopy

Duplicates of samples described above for WB and appropriate controls were simultaneously seeded onto 12 well tissue culture plates containing sterile round cover glasses. At 1 min, 30 min, 60 min, and 150 min post-infection, cells were washed, fixed with fresh 4% paraformaldehyde in PBS, rinsed in PBS, treated with 0.1% Triton X-100 in PBS for 5 min, rinsed, and processed for confocal microscopy with anti -mouse NF-κB P65Ab (Dil 1/100; Santa Cruz Biotech, CA) followed by Alexa 488 labeled secondary Ab and propidium iodine (Molecular Probes). Results are from 3–7 independent experiments (n = 2–3 slides per treatment). Confocal images were acquired with a Leica Laser Confocal microscope TCS-NP, using Leica software and identical parameters for all samples, namely, a 100X objective with a numerical aperture of 1.4 in a 1024×1024 format, a pinhole of 0.7 airy, and a Z section of 0.2 µm.

For colocalization studies of cruzain and NF-κB P65, macrophages infected for 30 min with WT *T. cruzi* and treated or not with K11777, or similarly infected with cruzain-deficient *T. cruzi* and treated or not with K11777, and non-infected controls were fixed and stained sequentially with rabbit anti-NF-κB P65 Ab (Dil 1/100; Santa Cruz Biotech) and Alexa 488-labeled secondary Ab, followed by rabbit polyclonal anti-cruzain antibody (Dil 1/100) [24] previously labeled with Alexa 594 according to manufacturer's instructions (Molecular Probes).

For confocal studies of IL12 expression, macrophages treated or not with K11777 were infected with WT or cruzain-deficient *T. cruzi* for 1 h (ratio 0.5 trypomastigotes/macrophage) followed by overnight incubation with fresh RPMI medium at 37°C. Cultures were then exposed to 150 ng/ml purified LPS for 2 h (Sigma), followed by 5 µg/ml BFA (Molecular Probes) for 1 h at 37°C. Appropriate controls were included in three independent experiments (n = 2 per sample). Slides were then fixed as above, and simultaneously stained with anti-IL12 antibody (1/100) (R&D Systems) [33] for 1 h followed by secondary Cy2-labeled secondary Ab (dil 1/1000) (Biomed) and propidium iodine (PI) (Dil 1/5000) (Molecular Probes). Coverslips were mounted with Vecta-Shield (Vector Lab). All slides were observed and documented on the same day in a Leica microscope, model TCS-NP, using Leica software and identical confocal parameters as described above. The microscopist analyzed all slides blindly. Fluorescence was then analyzed quantitatively with Openlab software (Improvision). Fluorescence intensity was recorded at random (n = 12 per sample) and statistically analyzed (t test).

For other additional studies, macrophages infected with WT and cruzain-deficient CA-I/KR, and uninfected controls were stained with a macrosialin (mouse CD68 homolog) specific Ab (Serotec) (Dil 1/200) followed by a CY2 affinity-pure donkey anti-rat IgG (Jackson Immuno-Research lab) secondary Ab and PI (Molecular Probes).

### Proteolysis of NF-κB P65

Human recombinant NF-κB P65 (rNF-κB P65) (Active Motif, Japan) expressed in *E. coli* from a full-length cDNA clone has a 14 aa deletion at the C-term. One µl of rNF-κB P65 was diluted to 150 nM in 10 µl of 1x buffer (with 5 µl 1M DTT/ml or 5 mM DTT) and reacted or not with recombinant cruzain at 150 nM (1/1) and serial dilutions (1/10 to 1/1000) in 100 mM Na Acetate Buffer pH 5.5 for 2 h at 37°C. Human rNF-κB P65 was also reacted with sonicates from 2.5×10^5^ wild type CA-I/72 or cruzain deficient CA-I/KR parasites as above. Samples were then resolved by WB as above with anti- NF-κBP65 Ab.

### *T. cruzi in vitro* assay

To better understand the effect of macrophage activation on parasite intracellular development, macrophages were infected with WT *T. cruzi* (ratio 0.5 trypomastigotes/cell) for 1 h at 37°C. Cultures included untreated controls, and macrophages treated with 150 nM purified LPS [29] as follows: LPS was added 1 h prior to *T. cruzi* infection, concomitantly with infection, or 1 h after infection. Cultures were treated with LPS for up to 48 h. Duplicate cultures were fixed with 4% paraformaldehyde at 1 h (t_o_), 24 h, and 48 h post-infection. Cells were stained with PI and the mean number of intracellular parasites/cell was estimated in 200 cells per slide (n = 3 slides/treatment) in two independent experiments [26]. Results were analyzed statistically (t test).

### Cruzain and L-arginase assays

Cruzain activity was determined in extracts of WT and cruzain-deficient CA-I/KR epimastigotes as previously described [22]. For L-arginase determinations [7], [34], J774 uninfected controls and macrophages infected with WT or cruzain-deficient trypomastigotes were used. Cells were collected 48 h post-infection and samples fixed in 4% paraformaldehyde for counting in a Coulter Counter Multisizer 3 (Beckman). Aliquots corresponding to 10^5^cells/50 µl were prepared by duplicate for L-arginase assays as described by Stempin et al. [7] Briefly, cells were lysed in 0.1% Triton X-100 buffer with protease inhibitors, centrifuged at 14,000 xg for 30 min at 4°C, and supernatants stored at −70°C. L-arginase activity in supernatants was determined as described [7], [34]. Urea (µg/ml) was measured at 540 nm. Results from three independent experiments (n = 3 per sample) were analyzed statistically with Prism 4 software.

## Results

### Cruzain-deficient *T. cruzi*

The cysteine protease inhibitor K11777 is cidal for WT *T. cruzi*. By exposing CA-I/72 *T. cruzi* to gradual step-wise micromolar increases in K11777 concentration over a period of 2 years, we generated a K11777-resistant and cruzain-deficient *T. cruzi* that retains negligible (<1%) protease activity as detected by a fluorescent protease substrate [22]. Both parental WT and cruzain-deficient parasites are clonal populations [22]. Cruzain-deficient *T. cruzi* have remained resistant to K11777 for 14 years even after passage through animals. The mechanism of drug resistance has been described in epimastigotes and is due to secretion of inactive, unprocessed cruzain [22]. The IC50 for CA-I/72 epimastigotes is 3–5 µM K11777 while cruzain-deficient epimastigotes are routinely maintained in 20-fold the lethal dose of K11777 (200 µM). IC50 values for intracellular wild type and cruzain-deficient amastigotes are 0.8–1 µM and 12 µM K11777, respectively.

Differing from parental CA-I/72 *T. cruzi* that are lethal at doses of ≥10^3^ trypomastigotes, cruzain-deficient parasites are unable to establish infection in normal mice even at doses of 10^6^ trypomastigotes and are only lethal in a severely immunodeficient Rag 1 −/− mouse model of Chagas' disease (Doyle, unpublished data). These results will be independently submitted for publication.

### An active site affinity tag identifies processed active proteases of wild type and cruzain deficient *T. cruzi*

**Figure 1:**
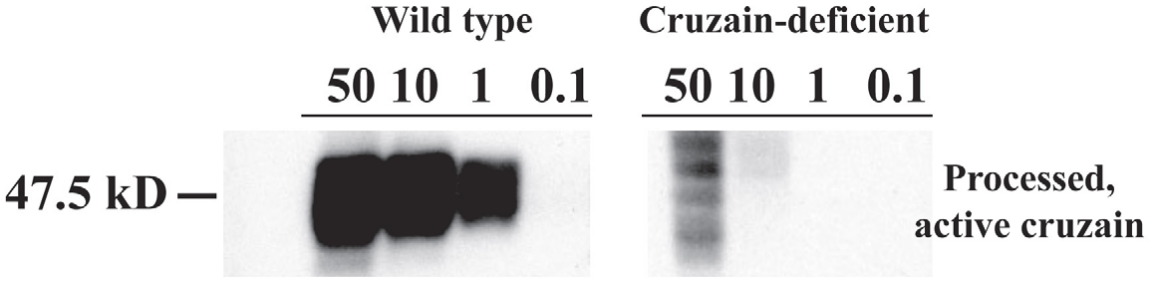
An active site affinity tag identifies processed active proteases of wild type and cruzain deficient *T. cruzi.* Iodinated DCGO4 (0.1, 1, 10, and-50 µM) was reacted with epimastigote extracts (10^5^ epimastigotes/lane). Processed, active cruzain (47.5 kD) was identified as the major protease in wild type *T. cruzi*. Higher molecular species identified as unprocessed cruzain by MALDI but no active protease were observed in cruzain-deficient parasites at 50 µM DCG04.

To identify and compare proteases in WT and cruzain-deficient *T. cruzi*, we used a functional proteomic method developed to profile protease targets in crude cellular extracts [27], [28]. The iodinated probe DCG04, allowed the specific identification of mature, active cruzain only in parental WT epimastigotes following SDS-PAGE (Figure 1). Cruzain-deficient parasites expressed unprocessed inactive cruzain with the prodomain attached as previously shown by WB [22] but no active mature protease. The identity of cruzain was confirmed by MS/MALDI and NCBI blast.

**Figure 2:**
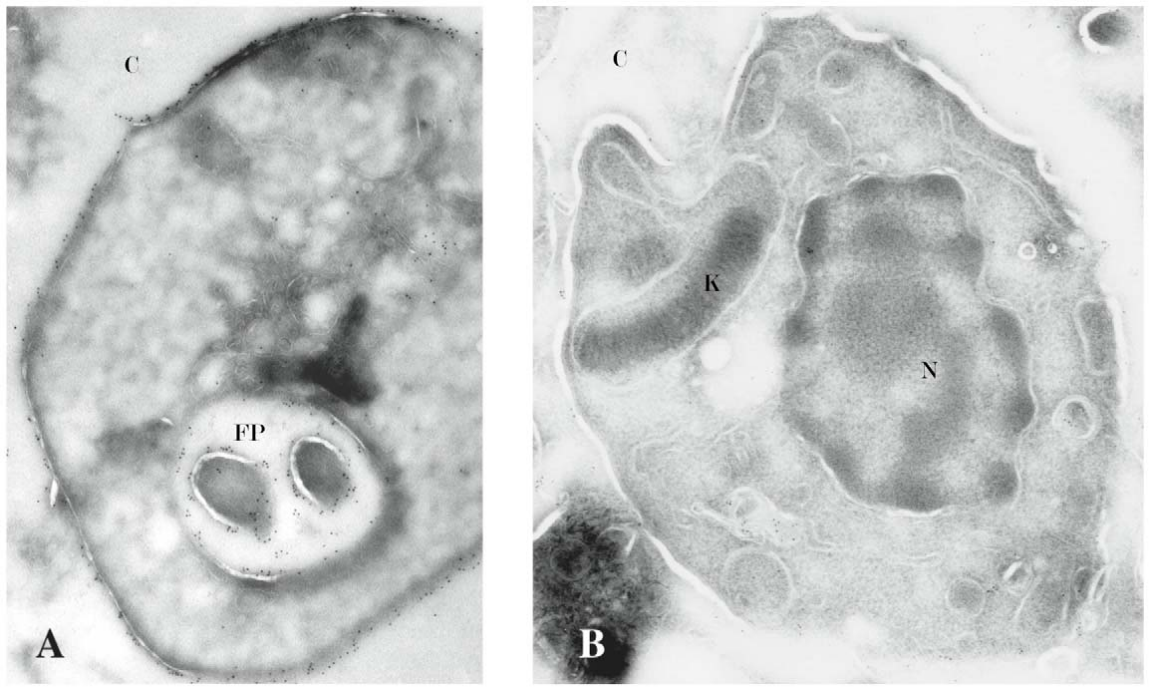
IEM confirms lack of surface-associated cruzain. A cruzain-specific gold-labeled secondary antibody identified cruzain on the cell surface of intracellular wild type and cruzain-deficient *T. cruzi* amastigotes. A 3-fold (p<0.1) reduction in gold-labeled Ab detection of cruzain was present on the cell surface and flagellar pocket of cruzain-deficient CA-I/KR (B) versus wild type amastigotes (A) (45,000X). Results are representative of three independent experiments. N, nucleus; K, kinetoplast; FP, flagellar pocket; C, cytoplasm of BESM cell.

To confirm decreased cruzain expression also in the intracellular pathogenic amastigote stage we performed comparative, quantitative IEM. A statistically significant (p<0.1) three-fold decrease in expression of membrane bound cruzain was confirmed for intracellular cruzain-deficient amastigotes as compared to WT controls (Figure 2).

### Transcription factor NF-κB P65 is only activated in macrophages infected with cruzain-deficient parasites

It has been proposed that proteases of *L. mexicana* cleave NF-κB to facilitate immune evasion [35]. We therefore assayed NF-κB activation in cells recently (≤60 min) infected by cruzain deficient parasites. We compared macrophage activation signaling pathways [36]–[39] as a consequence of early infection with WT versus cruzain-deficient *T. cruzi*. The infection of a cell population by *T. cruzi* follows a binomial distribution, with few cells very heavily infected while most cells remain non-infected. Moreover, infection with a high ratio of parasites per cell results in premature rupture of heavily infected macrophages. To prevent the premature release of intracellular parasites that may secondarily activate macrophages in the population, we used a very low infection ratio of 0.5 parasites per cell.

**Figure 3:**
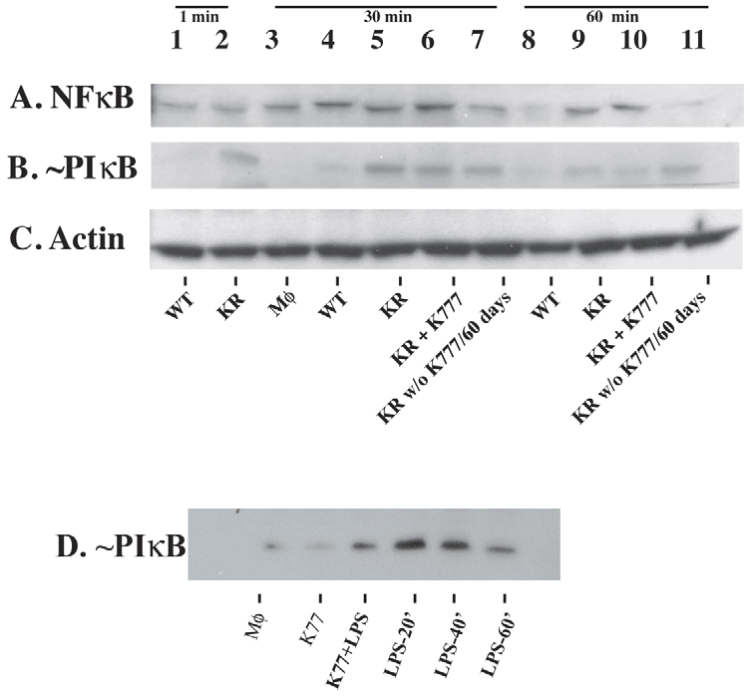
Cruzain-deficient (KR) but not WT parasites activate NF-κB. Uninfected controls and mouse macrophages (MΦ) infected with WT or KR *T. cruzi* were infected for ≤ 1 min (lanes 1, 2), 30 minutes (lanes 3–7) and 60 minutes (lanes 8–11). Cell extracts were western blotted with: **A**, anti-NF-κB P65 Ab; **B**, anti ∼P-iκB; **C**, anti-actin Ab. MΦ were infected as follows: lane #1, WT; lane #2, KR; lane #3, uninfected MΦ; lane #4, WT; lane #5, KR; lane #6, KR with 10 µM K11777; lane #7, KR cultured without K777 for 2 months; lane #8, WT*;* lane #9, KR; lane #10, KR with 10 µM K11777; lane #11, KR cultured without K11777 for 2 months. Results are representative of six independent experiments using two different methods. Controls (**D**) were MΦ treated or not with K11777 and/or LPS.

To detect macrophage activation, we performed WB targeting an NF-κB family member and its specific inhibitor [40]–[45]. Similar results were obtained with two methods and Abs from the two different vendors. WB analyses with anti-NF-κB P65 Ab (Figure 3A) showed expression in macrophages infected with WT *T. cruzi* at 1 min and 30 min post-infection (lanes 1, 4) was similar to uninfected controls (lane 3), and lower (lane 8) at 60 min post-infection. Phosphorylation of iκB is indicative of cell activation [40], [45]. Samples for WB were either heated to 70°C for 5 min (Santa Cruz Biotech. methods) or boiled for 10 minutes immediately prior to SDS-PAGE to dissociate complexes [30]–[32]. Samples blotted with specific anti ∼PiκB Abs from different commercial sources and using different protocols confirmed negligible ∼P iκB indicative of unresponsiveness in macrophages infected with WT *T. cruzi* for up to 60 min and uninfected controls (Figure 3B). In contrast, ∼P iκB indicative of activation was confirmed in macrophages infected with cruzain-deficient *T. cruzi* as early as 1 min post-infection. Additional macrophage controls infected with cruzain-deficient *T. cruzi* cultured for 2 months without K11777 showed intermediate results as these parasites only regained partial cruzain activity, and were not investigated further [22]. Additional controls were uninfected macrophages treated or not with LPS and/or K11777 (K77) (Figure 3D). In *L. mexicana* infected controls [32], NF-κB P65 degradation was still observed 48 h post-infection (data not shown).

### NF-κB is translocated to the nucleus only in macrophages infected with cruzain-deficient parasites

**Figure 4:**
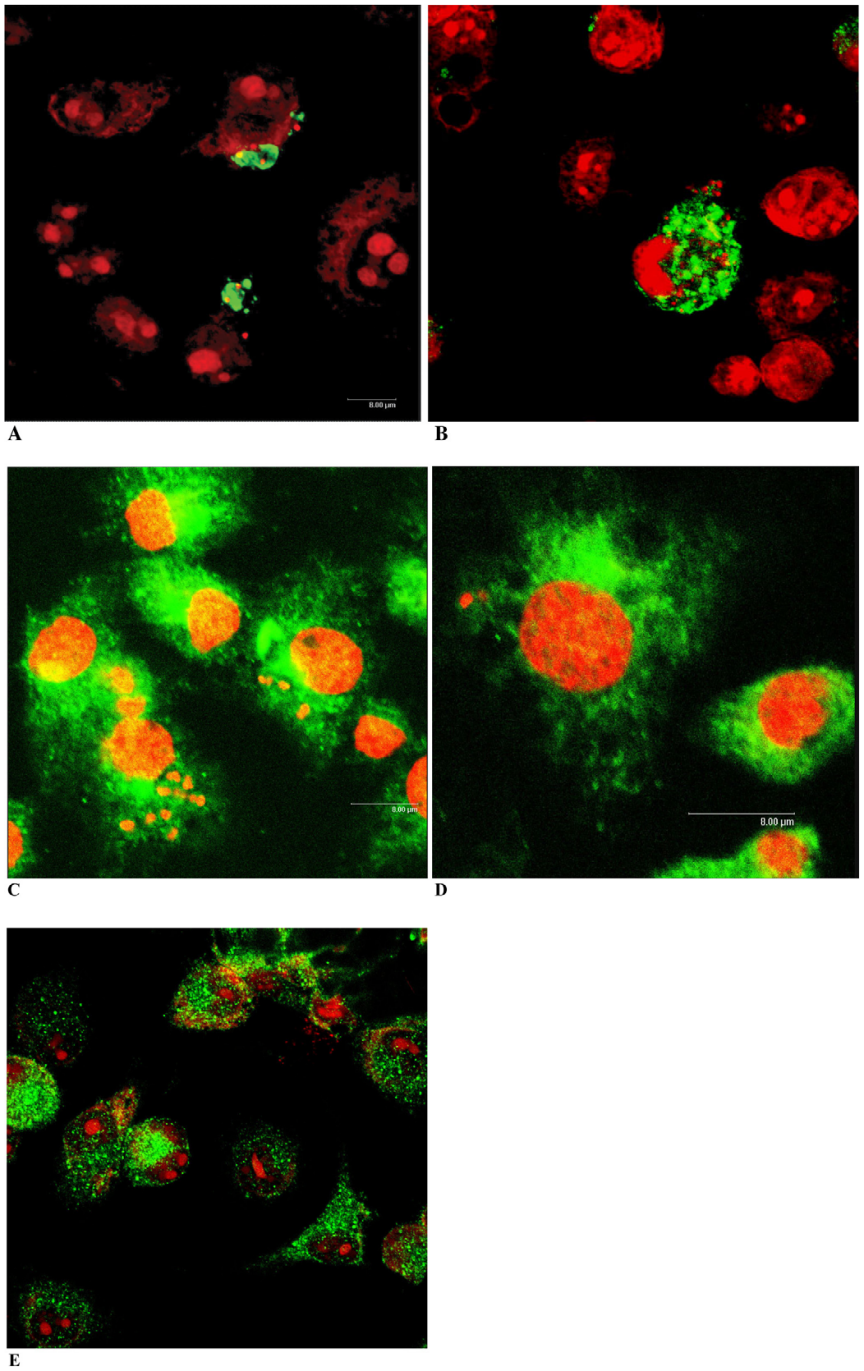
NF-κB P65 localizes to the cell surface of wild type parasites. Localization of NF-κB P65 (green fluorescence) was investigated in mouse macrophages. Parasite and host cell DNA was labeled with PI (red). Non-infected macrophages showed an evenly scattered distribution of cytoplasmic NF-κB P65 (E). Intracellular WT- *T. cruzi* appeared coated with macrophage NF-κB P65 (A, B). In contrast, abundant cytoplasmic (green fluorescence) and nuclear (yellow fluorescence) NF-κB P65 occurred in macrophages infected with cruzain deficient *T. cruzi* (C–D). Macrophages were infected for 30 min (A, C), and 60 min (B, D). Results are representative of four independent experiments.

To further investigate macrophage responses to WT versus cruzain deficient *T. cruzi*-infection, we performed confocal studies. NF-κB P65 localization was cytoplasmic in macrophage controls (Figure 4E). NF-κB P65 localization was also cytoplasmic in macrophages infected with WT *T. cruzi* for 1, 30, and 60 min post-infection confirming unresponsiveness to *T. cruzi* infection. Interestingly, NF-κB P65 covered the surface of WT amastigotes as early as 30 min post-infection (Figure 4A–B). Similar results were observed in BESM cells and thioglycolate elicited peritoneal mouse macrophages (data not shown). In contrast, intense NF-κB P65 label indicative of cell activation localized to the nucleus of macrophages infected with cruzain-deficient *T. cruzi* (Figure 4C–D) (yellow fluorescence), and no NF-κB P65 was seen bound to parasites for up to 60 min. Activation also occurred in macrophage controls infected with WT *T. cruzi* in the presence of 10 µM K11777 (data not shown).

### Colocalization of NF-κB and cruzain

**Figure 5:**
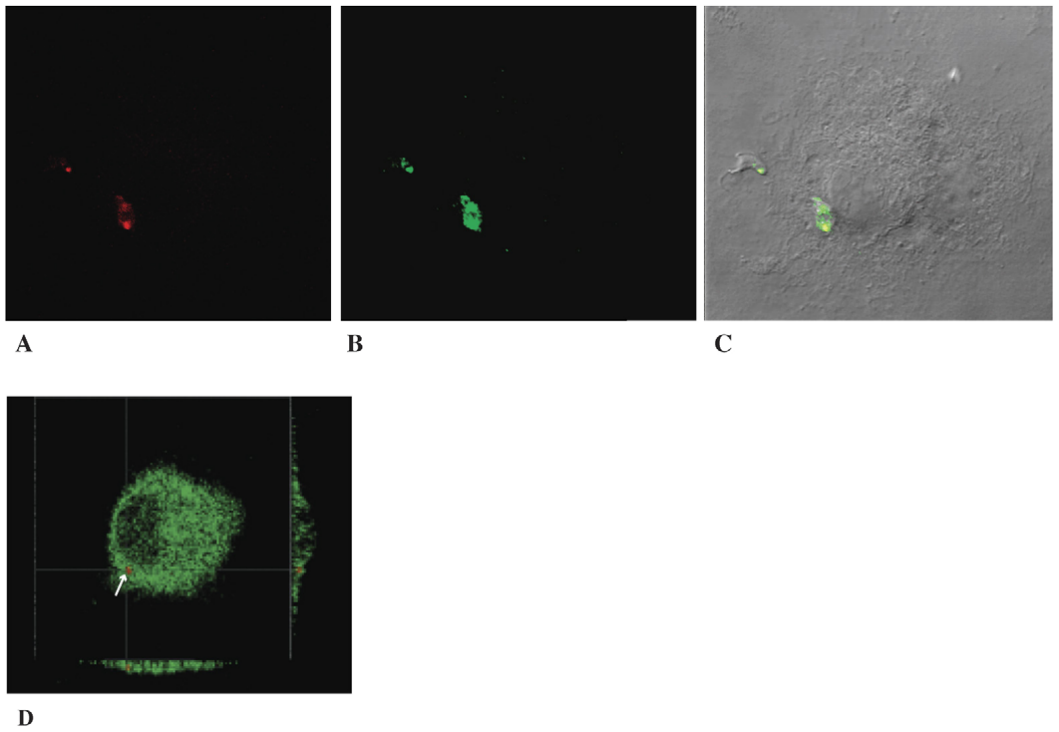
Colocalization of cruzain and NF-κB P65. **A–C.** Representative examples of colocalization of labeled cruzain (A) (red fluorescence) and NF-κB P65 (B) (green fluorescence) in the flagellar pocket region and/or cell surface of interiorized transforming parasites (C, merge). **D.** Lack of colocalization of accumulated cruzain (red fluorescence) and NF-κB P65 (green) in a macrophage infected as above with WT *T. cruzi* and treated with 10 µM K11777 that induces accumulation of unprocessed zymogen in the Golgi compartment (arrow) (22).

Colocalization of NF-κB P65 (green fluorescence) and cruzain (red fluorescence) on the cell surface of intracellular parasites occurred in macrophages infected with WT *T. cruzi* even during trypomastigote to amastigote transformation (Figure 5A–C). Intracellular WT *T. cruzi* treated with the trypanocidal inhibitor K11777 for 30 min showed marked cruzain accumulation presumably in the parasitic Golgi compartment [24] and no recruitment of NF-κB P65 to the parasite surface (Figure 5D, arrow).

### Proteolysis of human rNF-κB by cruzain

**Figure 6:**
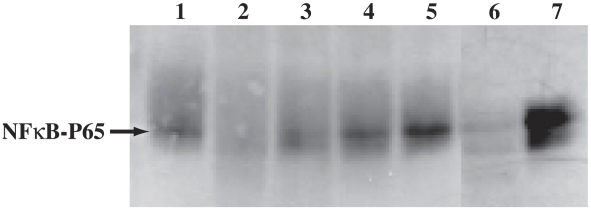
Proteolytic cleavage of r-human NF-κB P65 by wild type- and r-cruzain but not by proteases expressed by cruzain-deficient *T. cruzi.* Recombinant human NF-κB P65 was treated or not with cruzain or parasite extracts. Lane 1, untreated control; lane 2, r-cruzain (1∶1 molar ratio); lanes 3–4: dilutions 1∶10 and 1∶100 of r-cruzain, respectively; lane 5, r-cruzain (1∶1 molar ratio) and 10 µM K11777; lane 6, WT cruzain; lane 7, proteases in cruzain-deficient *T. cruzi*. Results are representative of two independent experiments.

Human rNF-κB P65 was proteolytically cleaved into two major fragments by native cruzain expressed by WT (Figure 6) but not by proteases expressed by cruzain-deficient *T. cruzi*. Similarly, recombinant cruzain degraded human r-P65 at equimolar concentrations under the experimental conditions used but not in the presence of 10 µM K11777.

### *T. cruzi* development in macrophages

**Figure 7:**
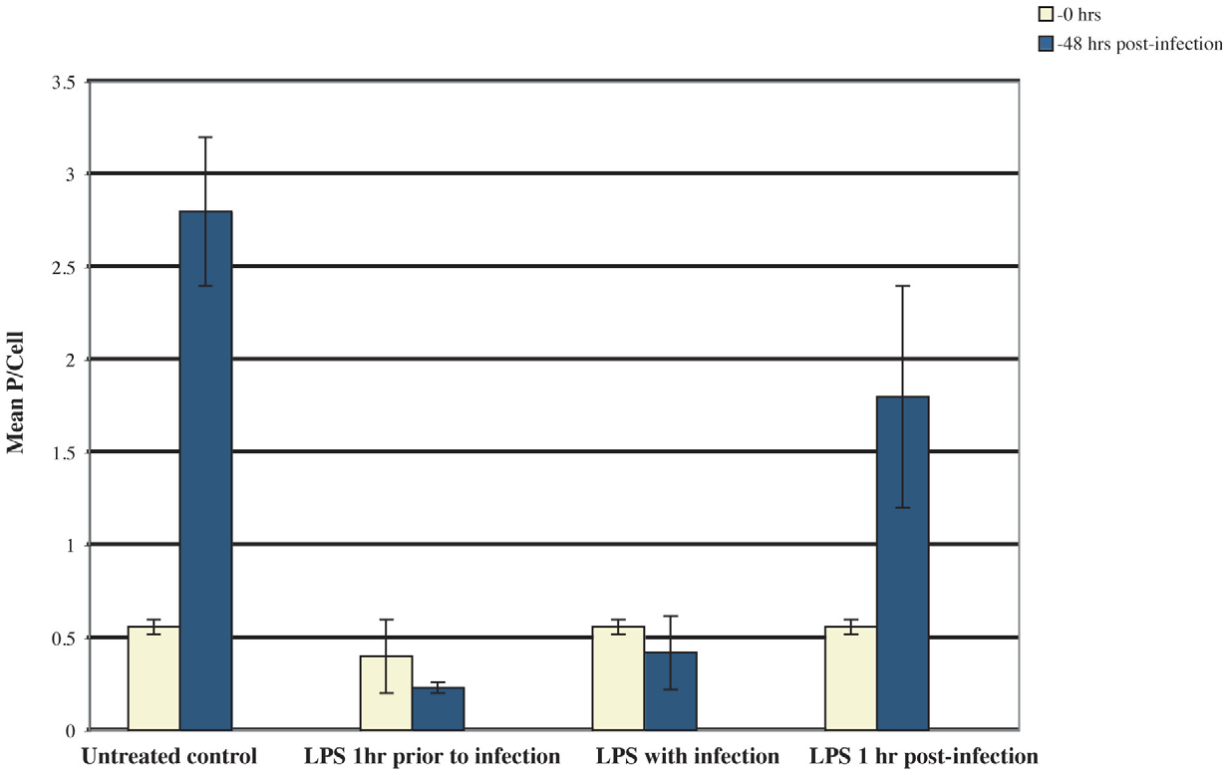
Macrophage activation prevents *T. cruzi* intracellular development. Mouse macrophages were infected with wild type *T. cruzi* and LPS was added as indicated. Mean parasites/cell were determined at 1 h and 48 h post-infection. Parasite death occurred when LPS was added prior to and concomitantly with *T. cruzi* trypomastigotes. Results are representative of two independent experiments (n = 3 per treatment).

To understand the effect of macrophage activation [37], [42] on *T. cruzi* intracellular survival and development, we performed quantitative *in vitro* assays [26]. *T. cruzi* divided normally in control macrophages and the mean number of parasites per cell (P/cell) increased from ∼0.5 at t_o_ to 2.8 at 48 h (Figure 7) [23]. Activation of macrophages with purified LPS 1h prior to or concomitantly with infection resulted in parasite death. *T. cruzi* developed well in macrophages treated with LPS 1h post-infection; the lower mean P/cell probably results from death of parasites still trapped within the parasitophorus vacuole when activation occurred. Thus macrophage unresponsiveness in early infection (<60 min) is crucial for parasite survival and intracellular development.

### IL12 expression

**Figure 8:**
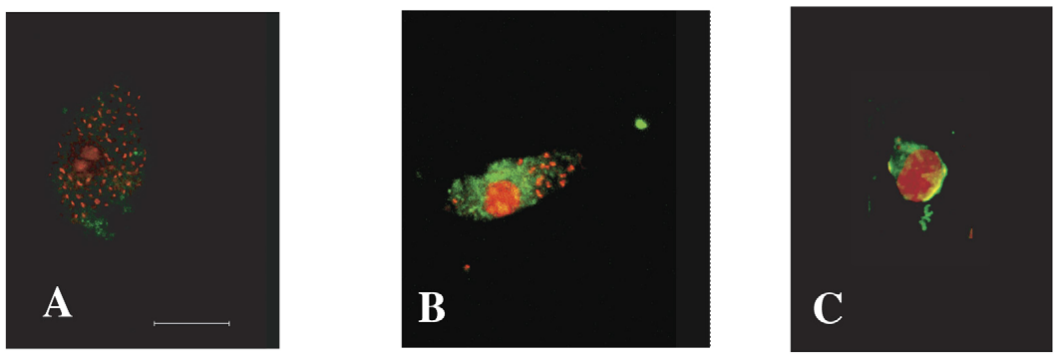
IL-12 expression. No significant IL12 expression was detected in macrophages infected with wild type *T. cruzi* while cells treated with 10 µM K11777 had significantly higher cytoplasmic IL12 levels. Increased IL12 expression also occurred in macrophages infected with cruzain-deficient *T. cruzi*. Results are representative of three independent experiments. **A.** Wild type-infected control. **B**. Macrophage infected wild type *T. cruzi* and treated with 10 µM K11777. **C.** Macrophage infected with cruzain-deficient *T. cruzi*.

To confirm macrophage unresponsiveness to infection with WT *T. cruzi* infection, we next investigated IL12 expression by confocal microscopy followed by fluorescence quantification with Improvision software. Macrophages infected with WT *T. cruzi*, and subsequently activated with LPS followed by BFA treatment, showed negligible cytoplasmic IL12 (Figure 8A). A significant increase (P<0.1) in IL12 accumulation occurred in macrophages infected with wild type *T. cruzi* and treated with the inhibitor K11777 that prevents cruzain activity (Figure 8B). Similarly, activation and significant IL12 accumulation occurred in macrophages infected with cruzain-deficient *T. cruzi* (Figure 8C).

### L- arginase assays

Other investigators have reported increased L-arginase activity induced by cruzain treatment of cells [7]. Only WT parasites induced 2-5 fold higher L-arginase activity in host macrophages while cruzain-deficient parasites failed to increase enzymatic activity (P<0.01). Representative values from one of 3 independent experiments are 3.13±0.02 µg urea/ml for macrophages infected with WT parasites, 1.56±0.02 µg urea/ml for macrophages infected with cruzain-deficient parasites, and 1.5±0.01 µg urea/ml for uninfected macrophages.

## Discussion

A complex and dynamic scenario of host cell-parasite interactions is becoming apparent as pathogens use different mechanisms to subvert host-cell signaling pathways or modulate their kinetics during intracellular development. Several reports show that *T. cruzi* modulates signaling pathways in mammalian cells [15], [43], [44]. Some authors propose that activation of NF-κB proteins may modulate tissue specificity, as muscle cells that preferentially harbor *T. cruzi* do not become activated [37] while others detect muscle and endothelial cell activation post-infection [45], [46]. Our interest was to investigate the role of the protease cruzain during early infection (<60 min) of macrophages by *T. cruzi,* and in particular on the nuclear factor NF-κB P65, using the inhibitor K11777 and phenotypic cruzain knockouts [22].

A major role of the NF-κB family is the regulation of aspects of the innate and adaptive immune responses. NF-κB members control the transcription of genes encoding cytokines and antimicrobial molecules, as well as genes regulating cell differentiation, survival, proliferation and apoptosis [38]. The complex response depends on the cell type and on the nature, duration and intensity of the activating signal. NF-κB complexes are normally inactive in the cytoplasm and upon activation they enter the nucleus to modulate gene expression. Membrane receptors (*e.g.* TRL) and other signaling regulators are also involved [38]–[42], [43]–[52].

To compare infection with WT versus cruzain-deficient *T. cruzi*, we performed WB of NF-κB P65 and ∼P iκB (Figure 3) [39], [47] that showed unresponsiveness of macrophages infected with WT parasites. Confocal studies showed that WT parasites rapidly sequestered NF-κB P65 onto their cell surface (Figure 4, A–B). Similar events occurred during early infection of peritoneal mouse macrophages and BESM cells (data not shown). Cruzain localized to the cell surface of WT intracellular amastigotes (Figure 2) [53] and colocalized with NF-κB P65 (Figure 5). Treatment with K11777 caused accumulation of unprocessed and inactive protease in the Golgi [24] and abrogated NF-κB P65 sequestration by the parasite (Figure 5D). Degradation of NF-κB P65 was noted 60 min after infection with wild-type *T. cruzi* (Figure 3) and native cruzain degraded recombinant nuclear factor (Figure 6). Moreover, inhibition of IL12 confirmed macrophage unresponsiveness in early WT *T. cruzi* infection (Figure 8A).

In contrast, macrophages infected with cruzain-deficient *T. cruzi* became rapidly activated via NF-κB P65 (Figure 3, KR) and intense nuclear localization of P65 occurred shortly after infection (Figure 4C–D) highlighting a role for cruzain in the modulation of host cell signaling pathways. As anticipated, infection with cruzain-deficient parasites induced IL12 accumulation (Figure 8C).

Preventing macrophage activation is crucial for a successful infection. Indeed, wild type *T. cruzi* were killed when macrophages were activated with LPS prior to or during infection (Figure 7), presumably while parasites are still contained within the parasitophorus vacuole [54]–[57] and are susceptible to cidal macrophage products. Other macrophage metabolic pathways were also modulated by *T. cruzi*. Infection with WT but not cruzain-deficient *T. cruzi* significantly increased L-arginase activity [6], [7]. A similar phenomenon occurs during *Leishmania* infection [58]. We also observed down-modulation of macrosialin, the murine analog of human CD68, in cells infected with WT *T. cruzi* (not shown). Further studies may identify other host cell molecules modulated by *T. cruzi* [59]–[63].

Experimental and clinical evidence shows a correlation between the severity of Chagas' disease and the persistence of *T. cruzi* within tissues [64]–[65]. NF-κB complexes are important for the development and function of both innate and adaptive immune responses triggered by pathogens [38], [51]. Metabolic pathways of the innate immune system induced in early infection have important consequences in the evolution of the disease as they play a role in the control of *T. cruzi* replication, tissue distribution and degree of parasitism. As infection progresses, an adaptive immune response develops with production of high levels of inflammatory cytokines, IFNγ and NO radicals [38], [59], [66]–[71]. In the case of *T. cruzi*, TLR2 and TLR9 receptors mediate cellular activation [43], [59], [66], [72]–[73].

Our results show that macrophages infected with WT *T. cruzi* remained unresponsive in early infection, and support the hypothesis that intracellular pathogens are uniquely protected from macrophage activation [38]. Confirming our results, expression profiling of bone marrow macrophages infected with *T. cruzi* revealed very few transcriptional changes during the first 12 hours. At 24 hours post infection, both macrophages and fibroblasts express some interferon-regulated genes [62], [74], [75]. The *L. mexicana* cruzain homologue, “cpb”, likewise degrades NF-κB P65, IκBα, and IκBβpreventing macrophage activation [35]. Similar strategies to suppress immune responses have been found in the coccidian parasite *Theileria* [76], and in poliovirus and other picornavirus as viral proteases cleave NF-κB P65 generating inactive products [77]. We then hypothesize that cruzain plays a key role by degrading NF-κB P65 and hindering activation of innate phagocytes recruited to the bite site [5]. Macrophage unresponsiveness would favor parasite survival in early infection and delay the onset of the immune response. A successful macrophage infection results in the production of hundreds of infectious *T. cruzi*. For example a single CA-I/72 parasite originates ∼130 infectious trypomastigotes in just 4.5 days [23]. Once the host immune response is triggered on [65]–[67], parasite development would be restricted to permissive tissues such as muscle cells [37], [71]. Thus cruzain may function during the early events of macrophage infection favoring immune evasion by *T. cruzi.*
